# Assessment of Risk Factors Correlated with Satellites and In-Transit Metastases in Primary Cutaneous Melanomas

**DOI:** 10.3390/medicina62061015

**Published:** 2026-05-23

**Authors:** Bianca Roxana Natarâş, Sorina Maria Tăban, Aura Jurescu, Octavia Cornelia Viţa, Remus Florin Cornea, Ioana Hurmuz, Adelina Vidac, Daciana Grujic, Valentin Tudor Popa, Alis Liliana Carmen Dema

**Affiliations:** 1 Anapatmol Research Centre, Victor Babeş University of Medicine and Pharmacy, 300041 Timişoara, Romania; 2 Department of Plastic Surgery, Victor Babeş University of Medicine and Pharmacy, 300041 Timişoara, Romania

**Keywords:** melanoma, satellites, in-transit metastases

## Abstract

*Background and Objectives:* This study aimed to identify the risk factors of primary cutaneous melanomas associated with microsatellites, satellites, and in-transit metastases. *Materials and Methods*: We performed a retrospective study on patients diagnosed with invasive primary cutaneous melanomas in two pathology departments. The cases were distributed into two groups, comparing the clinical–pathological features of cases that presented microsatellites, satellites, and in-transit metastases with cases that did not present microsatellites, satellites, and in-transit metastases. *Results*: From the total number of primary invasive cutaneous melanomas diagnosed (n = 204), 22% presented microsatellites, satellites, and in-transit metastases (n = 46). The presence of microsatellites, satellites, and in-transit metastases was strongly correlated with a Breslow index > 4 mm, a Clark level of IV or V, and a pT3 or pT4 pathological stage (*p* < 0.0001). Those cases were also associated with lymphovascular invasion (*p* = 0.0013), ulceration of the primary melanoma (*p* = 0.0037), and an increased mitotic rate (*p* = 0.0078). The presence of microsatellites, satellites, and in-transit metastases is associated with higher rates of lymph node metastases (*p* = 0.0006) and recurrence (*p* = 0.0282). *Conclusions*: The most important risk factors associated with microsatellites, satellites, and in-transit metastases are represented by a Breslow index > 4 mm, a Clark level of IV or V, and an advanced pathological stage.

## 1. Introduction

Invasive melanomas represent approximately 1% of all skin cancer cases, but they are accountable for more than 75% of skin cancer deaths [1]. The skin melanomas represent 1.7% of the global cancer diagnoses, according to GLOBOCAN [2]. In 2020, the global burden of melanoma increased to 325,000 cases from 230,000 cases in 2012, a 41% increase [3]. The age-standardized incidence rate is 3.8/100,000 for males and 3.0/100,000 for females, with cumulative lifetime risks of 0.42% and 0.33%, respectively [4]. If diagnosed in the early stages, melanoma has a high survival rate, approximately 94% [5].

Cutaneous melanoma metastases represent a relatively frequent event as the first manifestation of advanced disease or evidence of recurrence [6]. Most frequently, the skin metastases have origin in colon and breast cancer, but the incidence of cutaneous metastatic melanoma is continuously increasing, comprising 10–17% of patients affected by melanoma [6].

Microsatellite, satellite, and in-transit melanoma metastases (SITMs) represent types of non-nodal locoregional metastases that presumably happen as a result of intralymphatic or possibly angiotropic tumor extension [7]. They are clustered together, regardless of the number of lesions, as parameters of the N category in the 8th edition of the American Joint Committee on Cancer (AJCC) melanoma staging system [7,8]. The microsatellite definition is as follows: a microscopic cutaneous and/or subcutaneous metastasis adjacent to or deep to the primary melanoma and completely discontinuous from the primary melanoma, with the space between occupied by unaffected stroma. Satellites are defined as clinically evident cutaneous and/or subcutaneous metastases situated within 2 cm of the primary melanoma but discontinuous from the primary melanoma. In-transit metastases have a similar definition but are located more than 2 cm from the primary melanoma, in the area between the primary tumor and the regional lymph node basin. The presence of microsatellites, satellites, and in-transit metastases upstages patients to stage III [8].

According to current guidelines, the diagnosis of satellitosis and in-transit metastases without any additional lymph node spreading corresponds to stage IIIB disease, with a 5-year survival rate of 69% [9,10]. The involvement of the lymph nodes in the context of satellitosis and in-transit metastases is classified as stage IIIC, being associated with a 5-year survival rate of 49% [9,10]. It is crucial to separate satellitosis and in-transit metastases from distant cutaneous or subcutaneous metastases, which correspond to stage IV disease, presenting a 5-year survival of <30%. SITM is usually diagnosed in <20% of patients with cutaneous melanoma [11,12,13,14].

Patients with both satellites/in-transit metastases and lymph node metastases present a worse prognosis than patients with either satellites/in-transit metastases or nodal metastases alone [15,16]. Sentinel lymph node metastasis is one of the most important predictors of survival for patients with melanoma, and intravascular extension of tumor cells within lymphatics is widely accepted as a main mechanism of metastasis [17,18].

We analyzed microsatellites, satellites, and in-transit metastases together as SITM to examine their association with classic histologic factors (Breslow index, Clark level, ulceration, mitotic rate, lymphovascular invasion) as well as with lymph node metastases and recurrence patterns.

We observed that only a limited number of studies evaluated the clinical–pathological characteristics of melanomas that presented microsatellites, satellites, and in-transit metastases. The majority of the studies investigated either melanomas with microsatellites and satellites or melanomas with in-transit metastases.

### Aim

The main aim of this study was to investigate the clinical and pathological features associated with the presence of microsatellites, satellites, and in-transit metastases in invasive primary cutaneous melanomas in order to gain a better understanding of those cases.

Another goal was to investigate whether microsatellites, satellites, and in-transit metastases are correlated with the development of lymph node metastases and recurrence.

## 2. Materials and Methods

We obtained the ethics approval number, 90/19 December 2022, from the doctoral school of our university.

We performed a retrospective observational study, identifying all the cases with a primary invasive cutaneous melanoma histopathological diagnosis from January 2018 to December 2023 from the databases of two institutions in Western Romania.

Inclusion criteria: invasive primary cutaneous melanomas with or without microsatellites, satellites, and in-transit metastases with a complete histopathological report.

Exclusion criteria: cases of microsatellites, satellites, and in-transit metastases without the complete histopathological report of the primary cutaneous melanoma, in situ melanomas.

After case selection, we introduced the data in an Excel table that contained the clinical and pathological parameters of the primary invasive cutaneous melanomas.

We divided the cases into two groups:

- Primary invasive cutaneous melanomas that presented microsatellites, satellites, and in-transit metastases;
- Primary invasive cutaneous melanomas without microsatellites, satellites, and in-transit metastases.

We analyzed and compared the following clinical–pathological parameters: age, sex, primary tumor location, histological subtype, pathological stage, Breslow index, Clark level, mitotic rate, ulceration, regression, inflammatory infiltrate type (brisk/non-brisk), perineural invasion, lymphovascular invasion, SITM, lymph node status, and the recurrence pattern.

The resected specimens were placed in formalin, embedded in paraffin, and stained with hematoxylin–eosin stain in the two Pathology Departments.

### 2.1. IHC Analysis

For some of the cases, we used immunohistochemical (IHC) markers for the diagnosis. For the IHC investigation, additional sections from the selected paraffin blocks, with a thickness of 3–4 µm, were placed on Super Frost Ultra Plus slides. We used the following primary antibodies: Melan A (clone A103—DAKO, ready to use (RTU), S100 [polyclonal—DAKO, RTU], Sox 10 (clone EP268—Dako, RTU), and HMB45 (clone HMB45—DAKO, RTU). Antigen retrieval was performed by Heat-Induced Epitope Retrieval (HIER) in target retrieval solution pH6 (for S100 and Sox 10), pH9 (for Melan A), and enzymatic pretreatment for HMB45 for 20 min at 98 °C.

### 2.2. Statistical Analysis

The statistical analysis of the evaluated parameters was performed using the functions of Microsoft Office Excel 2010 (Microsoft Corp., Redmond, WA, USA) and GraphPad Prism software, Version 9.5.1 (GraphPad Software Inc., San Diego, CA, USA). To analyze the differences between various parameters, we used Fisher’s exact test. The results were considered statistically significant when the value of *p* was <0.05.

Statistical analyses for multivariable binary logistic regression were performed using IBM SPSS Statistics software, version 31 (IBM Corp., Armonk, NY, USA). Multivariable logistic regression models were constructed to evaluate if an increased Breslow thickness, a higher mitotic activity, lymphovascular invasion, ulceration, tumor location, and histologic subtype were independently associated with SITM. Multivariable logistic regression was also used to evaluate the association between SITM and lymph node metastases and recurrence.

Adjusted odds ratios (aORs) with 95% confidence intervals (CIs) were calculated for all variables. Statistical significance was defined as a two-sided *p* < 0.05.

The small number of recurrences precludes robust Kaplan–Meier or Cox modeling.

## 3. Results

A total of 204 patients were diagnosed in the two institutions with primary cutaneous melanomas between January 2018 and December 2023. The clinical and pathological features of those cases are presented in Table 1.

Multivariable logistic regression analysis demonstrated that increased Breslow thickness (OR 1.14, 95% CI 1.03–1.26, *p* = 0.009), higher mitotic activity (OR 1.11, 95% CI 1.02–1.21, *p* = 0.014), and presence of lymphovascular invasion (OR 5.09, 95% CI 1.50–17.23, *p* = 0.009) were independently associated with SITM. Ulceration, tumor location, and histologic subtype were not independently associated with SITM after adjustment. Odds ratios (ORs) with 95% confidence intervals (CIs) were calculated using maximum likelihood eSITMation—Table 2.

Twenty-two percent of patients presented microsatellites, satellites, and in-transit metastases. We compared the clinical–pathological features of the cases that presented microsatellites, satellites, and in-transit cutaneous metastases—46 cases (the first lot)—with the cases that did not present microsatellites, satellites, and in-transit metastases—158 cases (the second lot).

In 33 of the 46 cases (72%), the microsatellites, satellites, and in-transit metastases were diagnosed at the same time as the cutaneous melanoma (synchronous). In 13 cases (28%), microsatellites, satellites, and in-transit metastases were diagnosed after the primary cutaneous melanoma diagnosis, in a time frame that ranged from one month to 49 months, with a median time of 14.9 months (metachronous).

### 3.1. Sex and Age

From the 46 cases of melanomas with SITM, 23 were diagnosed in men (50%) and 23 in women (50%). From the 158 cases of melanomas without SITM, 85 were diagnosed in men (54%) and 73 in women (46%). The *p* value did not have statistical significance (*p* = 0.73). In both groups, the overwhelming majority of the patients were >40 years when they presented to the hospital with the primary cutaneous melanoma (96% in the first lot, 87% in the second lot). In the first group, the median age was 67.8 years. The median age of women was 67.9 years, and the median age of men was 67.6 years. In the second group, the median age was 58.7 years. The median age for women was 56.1 years, and the median age for men was 62.3 years.

### 3.2. Anatomical Location

Regarding the anatomical location of the primary tumor in the first lot, the majority of the cases were located on the trunk (48%), followed by the ones located on the upper extremities (22%), lower extremities (19%), and the head and neck area (11%). In the second lot, the majority of the cases were located on the trunk (40%), followed by the ones located on the lower extremities (23%), the head and neck area (20%), and the upper extremities (17%). The *p* value did not have statistical significance (*p* = 0.43).

### 3.3. Histological Subtype, Breslow Index, Clark Level, and Pathological Stage

Concerning the histological subtype in the first lot, 50% of the cases were diagnosed as superficial melanomas in the vertical growth phase, 43% as nodular melanomas, and 7% as acral melanomas. In the second lot, 57% of the cases were diagnosed as superficial spreading melanomas in the vertical growth phase, 26% as nodular melanomas, 9% as superficial spreading melanomas, and 8% as acral melanomas. The *p* value did not have statistical significance (*p* = 0.058).

In the first group, the majority of the cases presented a Breslow thickness > 4 mm (76%). A Breslow thickness between 2.01 and 4 mm and between 1.01 and 2 mm was observed in 13% and 11% of cases, respectively, while no cases had a Breslow thickness of less than 1 mm. In the second group, a Breslow thickness > 4 mm was also the most frequent category, accounting for 32.3% of cases. A Breslow thickness < 1 mm was identified in 28.5% of cases, followed by a Breslow thickness between 2.01 and 4 mm in 22.2% of the cases and between 1.01 and 2 mm in 17% of the cases. The *p* value was statistically significant (*p* < 0.0001).

In the first lot, only one case presented a Clark level of III (2%); the majority of the cases presented a Clark level of IV and V (98%). In the second lot, the majority of the cases presented a Clark level of IV and V (64%), and 36% of the cases presented a Clark level of II and III. The *p* value was statistically significant (*p* < 0.0001).

In the first group, the majority of the cases were pathologically staged pT3 or pT4 (89%), with only 11% of the cases being staged pT1 and pT2. In the second group, in the majority of the cases, a pT3 or pT4 pathological stage was assigned (54%), 46% of the cases being staged pT1 and pT2. The *p* value was statistically significant (*p* < 0.0001).

### 3.4. Lymphovascular Invasion, Perineural Invasion, and Mitotic Rate

Twenty percent of the cases in the first lot presented lymphovascular invasion, compared to 4% of the cases in the second lot. The *p* value was statistically significant (*p* = 0.0013). Nine percent of the cases from the first lot and 8% of the cases from the second lot presented perineural invasion. The *p* value was not statistically significant (*p* = 0.071).

Sixty-five percent of the cases in the first lot had a mitotic rate above 4 mitoses/mm^2^, 33% of the cases had a mitotic rate between 2 and 4 mitoses/mm^2,^ and only one case presented a mitotic rate of one mitosis/mm^2^. Forty-five percent of the cases in the second lot had a mitotic rate above 4 mitoses/mm^2^, 36% of the cases had a mitotic rate between 2 and 4 mitoses/mm^2,^ and 19% presented a mitotic rate of zero or one mitosis/mm^2^. The *p* value was statistically significant (*p* = 0.0078).

### 3.5. Tumor Infiltrating Lymphocytes (TILs), Ulceration, and Regression Status

The majority of the cases in both lots presented a non-brisk inflammatory infiltrate (72% in the first lot, 68% in the second lot). The *p* value was not statistically significant (*p* = 0.48).

The majority of the primary cutaneous melanomas in both groups were ulcerated (78% in the first group, 54% in the second group). The *p* value was statistically significant (*p* = 0.0037).

Regarding the regression status, 43% of the cases in the first lot and 41% of the cases in the second lot presented regression features. The *p* value was not statistically significant (*p* = 0.86).

### 3.6. Regional Lymph Node Status

Lymph node metastasis rates were calculated only among patients who underwent lymph node surgery (24/46 in the SITM group and 102/158 in the non-SITM group). In the first group, in 58% of the cases (14/24), an SNLB was performed, while in 42% (10/24) of the cases, a regional lymph node dissection was performed. In the second group, in 83% of the cases, an SNLB was performed (85/102), while in 17% (17/102) of the cases, a regional lymph node dissection was performed. In the first lot, in 16 of the 24 cases (67%), the lymph nodes were positive. From the 16 cases, 8 cases of lymph node metastases were diagnosed after the SITM diagnosis (50%), 6 were diagnosed at the same time as the SITM (37.5%), and 2 cases were diagnosed before the SITM diagnosis (12.5%). In the second lot, 26 of the 102 cases (25%) presented lymph node metastases. The *p* value was statistically significant (*p* = 0.0006)—Table 3.

While SITM shows a significant link to nodal involvement in an unadjusted, standalone environment, the effect disappears (*p* = 0.285) under multivariable control—Table 4.

### 3.7. Recurrence Pattern

**Figure 1:**
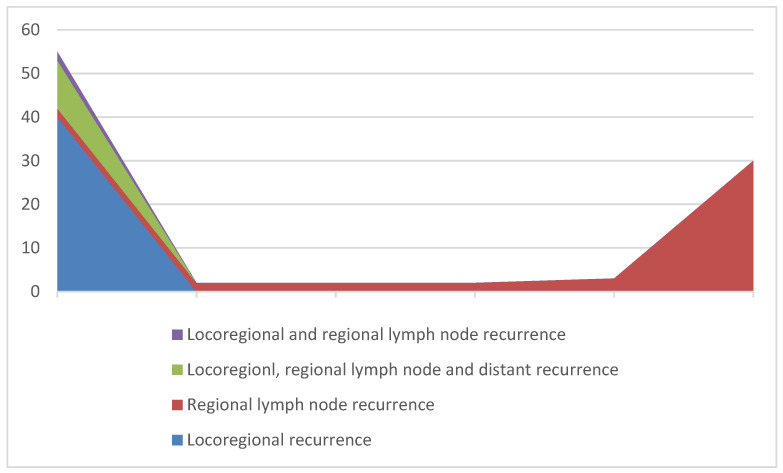
Time interval (months) for recurrences in the SITM group, based on recurrence type.

We considered the following types of recurrence: locoregional recurrence—represented by microsatellites, satellites, and in-transit metastases; regional lymph node recurrence; and distant recurrence—represented by distant skin metastases and extracutaneous metastases. In the first lot, 8 of the 46 cases (17%) presented another recurrence after the microsatellites, satellites or/and in-transit metastases diagnosis. In five of the eight cases the recurrence was present in the regional lymph nodes (62.5%); in one case, it was represented by locoregional recurrence (in-transit metastases); in one case, by regional lymph node recurrence and locoregional recurrence (in-transit metastases)—12.5%); and in one case, by regional lymph node metastases, in-transit metastases and distant metastases (cutaneous, subcutaneous and breast metastases—12.5%). The time period from the satellites or in-transit metastases diagnosis (in some of the cases, coinciding with the primary melanoma diagnosis) until the following recurrence diagnosis ranged from 2 months to 40 months (median time 11.5 months)—Figure 1. The majority of the cases (75%) presented one recurrence after the SITM diagnosis, one case presented two recurrences, and one case presented three recurrences.

In the second lot, 9 of the 158 cases recurred (6%). Five of the nine cases presented distant metastases (55.5%)—brain metastases (n = 2), small bowel metastasis (n = 2), and adrenal gland metastasis (n = 1). The rest of the cases (n = 4) presented loco-regional recurrence (44.5%): loco-regional lymph node recurrence (n = 3), and local recurrence (n = 1). The time period from the diagnosis of the primary cutaneous melanoma to the recurrence diagnosis ranged from 7 months to 62 months, with a median time of 28.5 months. All of the cases presented one recurrence in the time period that was evaluated. The *p* value was statistically significant (*p* = 0.0282)—Table 5.

The unadjusted association with recurrence may be partially explained by underlying tumor thickness and other adverse pathological features. Therefore, SITM did not remain an independent predictor of recurrence in the fully adjusted model—Table 6.

The recurrence analysis is limited by small event numbers and heterogeneous follow-up.

## 4. Discussion

In a study, 1.9% of the 1650 cutaneous melanoma cases evaluated presented SITM [19]. In-transit metastasis in cutaneous melanoma indicates a poor prognosis. The prevalence of this type of loco-regional metastasis varies greatly, fluctuating between 2.5% and 23% in different studies [20,21,22,23,24,25]. In our study, melanomas that presented SITM represented 22% of the cases, a much higher percentage compared to some studies but a similar percentage compared to other studies.

The majority of in-transit metastases in some studies were diagnosed within a year of the primary melanoma diagnosis or the first 3 years after the diagnosis [22,26]. In our study, the time interval from the primary cutaneous melanoma diagnosis to the satellites and in-transit metastases diagnosis varied between one month and 49 months.

In the literature, it is noted that until the age of 50 years, the incidence of melanoma in female patients is double compared to the male patients. By the age of 60, the incidence in male patients doubles, and by the age of 70, it triples compared to females [27]. In other studies, the females were diagnosed more often with melanomas that presented SITM (62.5% females, 37.5% males) [19]. In our study, the incidence of melanomas with SITM was the same in men and women, differing from other studies.

The median age for the patients diagnosed with SITM in the article by Weide and colleagues (66 years) is similar to the median age of the first lot of our study [28]. The median age was higher in the first group, with almost a decade (67.8 years), compared to the second one (58.7 years). Although in the first group, there were no significant differences between the median age in both sexes (67.9 years for women and 67.6 years for men), in the second group, the median age for women was significantly lower (56.1 years) than for men (62.3 years).

Huibers and colleagues concluded that the lower extremity is the most common site of the primary melanoma in patients with in-transit metastases (43.2%) [29]. Those results differ from our study, where the trunk was the most frequent site involved.

In some studies, the nodular subtype was described to be the most frequent subtype associated with satellites and in-transit metastases [30,31]. In our study, the results were different. The superficial spreading melanomas, in the vertical growth phase, represented the main histological subtype associated with SITM.

The depth of invasion (Breslow index) as a prognostic factor was described by Alexander Breslow in 1970, who was able to demonstrate a correlation between melanoma thickness and the risk of recurrence and metastasis [32,33]. Breslow thickness is one of the most influential parameters in the staging of cutaneous invasive melanomas [7]. A Breslow index > 4 mm was correlated with a high risk of developing in-transit metastasis in other studies [31]. In the first group, the percentage of cases that had a Breslow index > 4 mm was significantly higher compared to the second group. A very significant difference was also observed regarding the melanomas with a <1 mm Breslow index—absent in the first group compared to the second group.

The Clark level, an indicator of invasion of the tumor through the different skin layers, has historically been associated with prognostic significance for primary melanoma, with a worse prognosis in melanomas with an increased Clark level [34]. Clark defined the following five anatomic levels: I—no dermal invasion; II—the tumor cells invade the papillary dermis; III—the tumor cells are located at the border of the papillary dermis with the reticular dermis; IV—the tumor cells invade the reticular dermis; and V—the tumor cells invade the subcutis [34]. In other studies, a high Clark level is associated with satellites and in-transit metastases [30]. This observation corresponds to our study, where the overwhelming majority of the melanomas with SITM presented a Clark level of IV and V.

In other studies, the lymphovascular invasion was associated with the presence of satellites in 29% of the cases [35]. We obtained similar results (20% of the SITM cases presented lymphovascular invasion).

The mitotic index represents the number of mitotic figures in one mm^2^ of the infiltrative component of the tumor, evaluated on a routine hematoxylin and eosin (H&E) stain. Mitotic rate is considered an independent predictor of melanoma survival in multivariate analysis [36]. An important difference was noted for cases that presented >4 mitoses/mm^2^: in the first group, the percentage was higher compared to the second group.

The lymphocytic infiltrate can be classified as absent, brisk, or non-brisk [37]. Non-brisk inflammatory infiltrate represents the infiltrate distributed only focally and not along the entire base of the invasive component. Brisk infiltrate is defined by lymphocytes that infiltrate across the entire base of the vertical growth phase or infiltrate the entire invasive component diffusely [37,38]. Despite many studies affirming that a brisk lymphocytic response affects prognosis in a favorable way [39,40,41,42], other studies have shown this to be untrue [43]. In the two groups of our study, the majority of cases presented an inflammatory infiltrate of non-brisk type.

Ulceration has been included in the TNM classification of malignant melanoma since 2009, representing another essential prognostic factor, upstaging the tumor [9]. In another study, 52% of the cutaneous melanomas that presented satellites and in-transit metastases were ulcerated [30]. In our study, the percentage of the melanomas with SITM that were ulcerated was much higher—78%.

Although a standardized criterion for reporting histopathological regression in melanomas is lacking, it generally consists of a variable decrease in the dermal invasive melanoma cells, together with dermal fibrosis, melanophages, increased vascularity, inflammatory infiltrate, and epidermal attenuation [44]. Some studies suggested that regression is linked with a worse prognosis because it can contribute to the reduction in the Breslow thickness of the primary melanoma (when the deepest-located melanoma cells are absent). Other studies affirmed that when regressive aspects are present, it implies better survival, because the basis of regression is thought to be the host immune system activation against the tumor [44,45]. We did not observe significant differences regarding the presence of regression in the two groups from our study.

The involvement of the regional lymph node in metastatic disease represents an important outcome predictor in melanomas, being associated with a 50% decrease in survival compared to patients without lymph node metastases [46,47]. In a study conducted by Vita and colleagues, 66.6% of the melanomas with satellites and in-transit metastases presented lymph node metastases, showing that those cases present a more aggressive behavior and higher metastatic risk [48]. This percentage was similar to the one obtained in our study for the SITM group (67%).

In some studies, about half of all patients treated for melanoma presented recurrences [49,50]. In various studies, approximately 50% of the recurrences were present in the regional lymph nodes, 20% represented local recurrences, and 30% developed at distant sites [51,52,53]. In our study, the time interval from the satellites and in-transit metastases diagnosis until the following recurrence was shorter compared to the recurrence after the diagnosis of the primary melanoma in the second group. In the SITM group, the recurrence rate was higher compared to the second group.

A distinctive feature of our cohort was the predominance of thick and advanced melanomas in patients with satellites and in-transit metastases, together with high rates of nodal involvement and recurrence. This recurrence pattern may have practical implications for follow-up intensity, imaging surveillance, and treatment planning, particularly regarding earlier referral for multidisciplinary evaluation and systemic therapy consideration.

### 4.1. Diagnosis

Preferentially expressed antigen in melanoma (PRAME) is a tumor-associated antigen that has the potential to differentiate between melanocytic nevi (negative) and melanoma (positive). PRAME showed diffuse nuclear immunoreactivity (>75%) in 80–83.2% of primary melanomas and 80–87% of metastatic melanomas [54,55].

The molecular diagnostics that are used nowadays for the diagnosis of melanomas include fluorescence in situ hybridization (FISH), gene expression profiling (GEP), and comparative genomic hybridization (CGH), which recognizes chromosomal copy number variations, including multiplications or deletions of chromosomal segments [56]. Early CGH studies stated that more than 95% of melanomas presented chromosomal number, as opposed to only 13% of melanocytic nevi [57,58].

### 4.2. Treatment

#### 4.2.1. Surgical Treatment and Chemotherapy

First-line treatment for one or a few in-transit metastases is surgical excision. If the disease progresses or if excision is not an option, various other locoregional treatments are available, like intralesional or topical drug administration, cryotherapy, electrocoagulation, electrochemotherapy, radiotherapy, laser therapy, isolated limb infusion, and isolated limb perfusion [59,60].

Chemotherapy uses cytotoxic drugs to destroy melanoma cells that have extended beyond the skin, having limited effectiveness and causing side effects. In melanoma treatment, paclitaxel, dacarbazine, temozolomide, nab-paclitaxel, cisplatin, and carboplatin are commonly used chemotherapeutics [61].

#### 4.2.2. Targeted Therapy and Immunotherapy

Approximately 70% of melanomas have activating mutations in the MAPK pathway, two-thirds affecting the *BRAF* gene, representing a target for BRAF inhibitors (BRAFi), including dabrafenib, vemurafenib, and encorafenib. When those are used in combination with MEK inhibitors (MEKi)—trametinib, cobimetinib, or binimetinib—the 5-year survival for BRAF inhibition ranges from 31 to 35% [62,63,64].

After a complete surgical resection, immunotherapy can be administered as an adjuvant in patients who present a high risk of relapse or as a treatment for advanced melanomas [65].

The CheckMate 238 study, which included patients with stage IIIB-C or IV melanoma, demonstrated that adjuvant nivolumab had superior effects compared to ipilimumab regarding relapse-free survival and distant metastasis-free survival [66].

#### 4.2.3. Experimental Treatment

Animal models are considered a pivotal tool for a better understanding of the various aspects of human illnesses, including cancer. In vivo models offer more realistic information compared to in vitro experiments [67]. A research study proved that mice with melanoma that were treated with doxorubicin-loaded leukosomes displayed the highest reduction in tumor volume compared to untreated mice and mice treated with free doxorubicin [68].

### 4.3. Study Limitations

The main limitations are represented by the retrospective design of the study and the lack of survival analyses. Another limitation is represented by the small number of patients who presented microsatellites, satellites, and in-transit metastases.

## 5. Conclusions

The parameters that had the strongest correlation with the presence of microsatellites, satellites, and in-transit metastases were represented by the Breslow index, Clark level, and pathological stage (*p* < 0.0001), followed by lymphovascular invasion (*p* = 0.0013), ulceration of the primary melanoma (*p* = 0.0037), and the mitotic rate (*p* = 0.0078). Multivariable logistic regression analysis demonstrated that increased Breslow thickness, higher mitotic activity, and the presence of lymphovascular invasion were independently associated with SITM. Ulceration, tumor location, and histologic subtype were not independently associated with SITM after adjustment.

The presence of microsatellites, satellites, and in-transit metastases is associated with higher rates of lymph node metastases (*p* = 0.0006) and recurrence (*p* = 0.0282) but does not remain an independent predictor after adjustment for other adverse tumor characteristics. We emphasize the fact that patients diagnosed with melanomas require long-term surveillance to detect recurrences.

## Figures and Tables

**Table 1:** The clinical and pathological features of primary cutaneous melanomas.

		with SITM	with SITM	Without SITM	Without SITM	
		n = 46	%	n = 158	%	
Sex	Female	23	50	85	54	
	Male	23	50	73	46	*p* = 0.73
Age of diagnosis	≤40 years	2	4	20	13	
	>40 years	44	96	138	87	*p* = 0.174
Anatomic location	Head and neck	5	11	31	20	
	Upper extremities	10	22	27	17	
	Lower extremities	9	19	37	23	
	Trunk	22	48	63	40	*p* = 0.43
Histologic subtype	Superficial spreading	0	0	14	9	
	Superficial spreading, in the vertical growth phase	23	50	90	57	
	Nodular	20	43	41	26	
	Acral	3	7	13	8	*p* = 0.058
Breslow index	<1 mm	0	0	45	28.5	
	1.01–2 mm	5	11	27	17	
	2.01–4 mm	6	13	35	22.2	
	>4 mm	35	76	51	32.3	*p* < 0.0001
Clark level	II–III	1	2	57	36	
	IV–V	45	98	101	64	*p* < 0.0001
pT	pT1–pT2	5	11	73	46	
	pT3–pT4	41	89	85	54	*p* < 0.0001
Lymphovascular invasion	Absent	37	80	152	96	
	Present	9	20	6	4	*p* = 0.0013
Perineural invasion	Absent	42	91	146	92	
	Present	4	9	12	8	*p* = 0.071
Mitotic rate	0–1/mm^2^	1	2	30	19	
	2–4/mm^2^	15	33	57	36	
	>4/mm^2^	30	65	71	45	*p* = 0.0078
Inflammatory infiltrate	Brisk	13	28	51	32	
	Non-brisk	33	72	107	68	*p* = 0.48
Ulceration	Absent	10	22	72	46	
	Present	36	78	86	54	*p* = 0.0037
Regression	Absent	26	57	93	59	
	Present	20	43	65	41	*p* = 0.86

**Table 2:** Multivariable logistic regression for predictors of SITM.

Variable	OR	95% CI	*p* Value
Breslow thickness (per mm)	1.14	1.03–1.26	0.009
Mitotic rate (per mitosis/mm^2^)	1.11	1.02–1.21	0.014
LVI			
Absent	Ref.	—	—
Present	5.09	1.50–17.23	0.009
Ulceration			
Absent	Ref.	—	—
Present	0.96	0.37–2.51	0.929
Location			
Head and neck	Ref.	—	—
Inferior extremity	1.83	0.47–7.12	0.382
Superior extremity	2.65	0.69–10.12	0.155
Trunk	2.58	0.80–8.36	0.113
Histologic subtype			
Acral/lentiginous	Ref.	—	—
Nodular	1.22	0.26–5.71	0.805
Superficial spreading	1.31	0.29–5.92	0.723

**Table 3:** Lymph node metastases status.

		N = 24	%	N = 102	%	
Lymph node metastases	Absent	8	33	76	75	
	Present	16	67	26	25	*p* = 0.0006

**Table 4:** Multivariable logistic regression for lymph node metastases.

Covariate	Adjusted Odds Ratio (aOR)	95% Confidence Interval (CI)	(*p*)-Value	Statistical Status
SITM (Present vs. Absent)	1.86	0.60–5.80	0.285	Not Significant
Ulceration (Present vs. Absent)	1.92	0.77–4.80	0.161	Not Significant
Breslow Thickness (per mm)	1.13	0.94–1.36	0.198	Not Significant
Mitotic Rate (\1 mm^2^)	1.03	0.92–1.15	0.573	Not Significant
LVI (Present vs. Absent)	1.35	0.25–7.33	0.726	Not Significant
Location: Inferior Extremities	0.92	0.20–4.15	0.915	Not Significant
Location: Superior Extremities	0.78	0.16–3.83	0.760	Not Significant
Location: Trunk	1.95	0.50–7.68	0.339	Not Significant
Subtype: Nodular	1.42	0.31–6.58	0.655	Not Significant
Subtype: Superficial Spreading	0.63	0.16–2.59	0.526	Not Significant

**Table 5:** Recurrence status.

		N = 46	%	N = 158	%	
Recurrence	Absent	38	83	149	94	
	Present	8	17	9	6	*p* = 0.0282

**Table 6:** Multivariable logistic regression for recurrence.

Variable Component	Odds Ratio (aOR)	95% Confidence Interval (CI)	(*p*)-Value
Intercept	0.025	(0.003, 0.176)	(<0.001)
Breslow Thickness (per mm)	1.145	(1.036, 1.266)	0.008
Mitotic Rate (per mm^2^)	1.114	(1.024, 1.212)	0.012
LVI (Present vs. Absent)	5.157	(1.525, 17.444)	0.008
Ulceration (Present)	0.944	(0.361, 2.472)	0.907
Location: Inferior Extremities	1.857	(0.477, 7.228)	0.372
Location: Superior Extremities	2.685	(0.701, 10.278)	0.149
Location: Trunk	2.572	(0.797, 8.298)	0.114
Subtype: Nodular	1.172	(0.248, 5.540)	0.841
Subtype: Superficial Spreading	1.308	(0.288, 5.948)	0.728

## Data Availability

All data generated or analyzed during this study are included in this published article and can be provided if needed or requested by the reviewer.
